# Conservative Management of Phlegmon Appendicitis in Pregnancy: A Case Report

**DOI:** 10.7759/cureus.100969

**Published:** 2026-01-07

**Authors:** Hattan Degastani, Abdulrahman Alfadhel, Ali H Alsharedah, Tahir Mubarak, Jumanah Hamed Alqurashi

**Affiliations:** 1 Obstetrics and Gynecology, King Abdulaziz Medical City, Riyadh, SAU

**Keywords:** appendicitis, conservative, phlegmon appendicitis, pregnancy, uncomplicated appendicitis

## Abstract

Acute appendicitis is the most common non-obstetric surgical emergency mandating immediate intervention. The standard management of complicated appendicitis is surgical intervention; however, phlegmonous appendicitis has been sparsely studied in pregnancy. We report a case of phlegmonous appendicitis in pregnancy that was successfully managed with antibiotic therapy and close clinical and radiological follow-up, resulting in favorable maternal and fetal outcomes without the need for surgical intervention.

## Introduction

During pregnancy, the most prevalent cause of acute abdomen necessitating surgical intervention not related to pregnancy is acute appendicitis [1-7]. The reported incidence rate of acute appendicitis in pregnancy is 1:1,500, with higher prevalence in the second trimester [8-15]. The approach to acute appendicitis during pregnancy poses unique challenges and requires high clinical suspicion due to the anatomical and physiological alterations in pregnancy and increased maternal and fetal mortality [16-19]. The management options for uncomplicated acute appendicitis in the general population include surgical and conservative management. In uncomplicated acute appendicitis, conservative management using intravenous antibiotics was found to be comparable to surgical intervention in randomized clinical trials [19-21]. Optimal management is still a question of debate with regard to uncomplicated acute appendicitis. Laparoscopic appendectomy is considered the standard treatment of choice [19-25]. Recent studies have proven that conservative management can be a feasible option, avoiding surgical complications and allowing for a faster recovery [21-26]. A thorough understanding of the clinical course, epidemiology, and outcomes is needed to establish optimal management.

## Case presentation

We report a case of a 27-year-old primigravida at 31 + 5 weeks of gestational age with unremarkable antenatal care. She initially presented to the emergency department with sudden-onset right flank pain that had begun five hours prior to her presentation. The pain was not associated with nausea, vomiting, dysuria, or changes in bowel habits. Her cardiac and respiratory exams were unremarkable. Her abdominal exam revealed a soft, lax abdomen with no tenderness, a gravid uterus measuring 31 cm in fundal height that corresponds to her gestational age, and initial laboratory investigations, including urinalysis, which were within normal limits and showed no signs of urinary tract infection. She was managed conservatively with intravenous hydration and acetaminophen, resulting in symptomatic improvement. A urine culture was obtained, and the patient was subsequently discharged in stable condition.

Two days later, the patient re-presented to the emergency department with a recurrence of the same pain. At that time, her vital signs, physical exam, and laboratory workup remained unremarkable, while the urine culture result was pending. The patient was discharged in stable condition with a seven-day course of oral cefuroxime 500 mg, twice daily, for empiric treatment of urinary tract infection.

Ten days after the initial presentation, the patient returned with persistent right-sided abdominal pain with no associated symptoms. On examination, she was hemodynamically stable, with normal cardiac and respiratory exams. Her abdominal exam revealed a soft, lax abdomen with tenderness noted in the right upper quadrant, and Murphy's sign was elicited. Cardiotocography revealed normal fetal heart tracing with no contraction. Laboratory workup revealed leukocytosis with neutrophil predominance and elevated sedimentation rate (Table 1). Her urine culture was negative. Abdominal ultrasonography demonstrated a distended gallbladder containing multiple gallstones without wall thickening or pericholecystic fluid. The common bile duct was normal in caliber, measuring 0.3 cm. These findings were consistent with cholelithiasis without evidence of acute calculous cholecystitis. Consequently, the patient was admitted for further management and urgent consultation with the general surgery team.

**Table 1:** Initial investigations.

Test name	Result	Normal range
White blood cells	17.90/L	4-11/L
Neutrophils	14.30/L	7.5-10/L
Sedimentation rate	120 mm/hr	0-20 mm/hr

**Figure 1:**
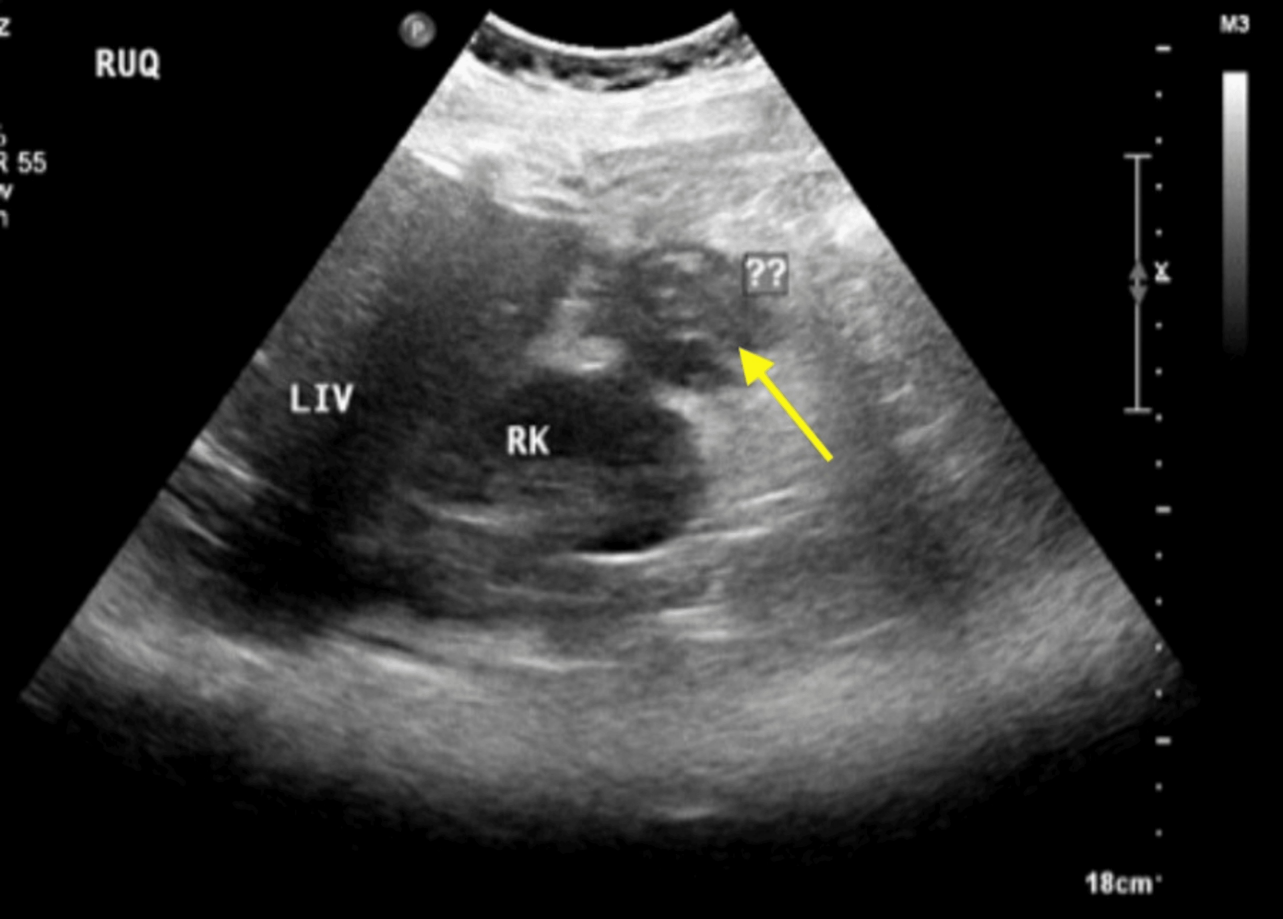
Ultrasonography of the abdomen. Ultrasonography of the abdomen demonstrating the ill-defined anechoic to hypoechoic structure adjacent to the right hepatic lobe with internal echogenicity.

During admission, the patient started on empirical intravenous piperacillin-tazobactam (Tazocin) at a dose of 4.5 g every six hours. A repeated abdominal ultrasonography (Figure 1) demonstrated an ill-defined anechoic to hypoechoic structure adjacent to the right hepatic lobe with internal echogenicity. These findings were non-specific, and acute appendicitis with possible complications could not be excluded. No renal calculi or hydronephrosis was identified.

**Figure 2:**
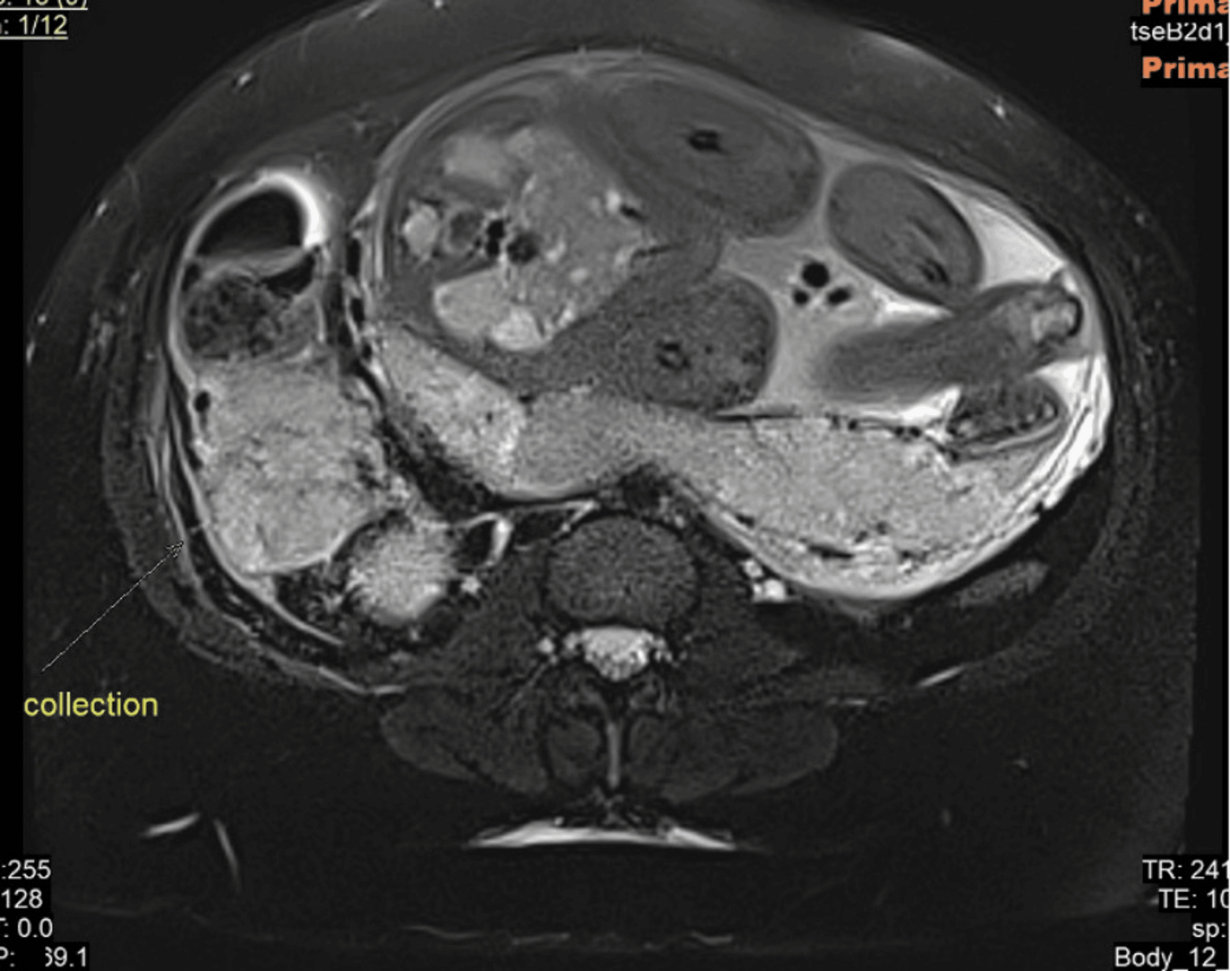
Magnetic resonance imaging of the abdomen. Magnetic resonance imaging of the abdomen revealing the subhepatically located appendix that was dilated to approximately 1 cm and associated with a large subhepatic phlegmon measuring 5.7 × 4.2 cm, along with small, tiny non-drainable abscess pockets.

To further evaluate the appendix, a pelvic magnetic resonance imaging (MRI) study was performed. MRI revealed a subhepatically located appendix that was dilated to approximately 1 cm and associated with a large subhepatic phlegmon measuring 5.7 × 4.2 cm, along with small, tiny non-drainable abscess pockets. Reactive thickening of the peritoneal reflection was noted, as well as reactive inflammatory changes involving hepatic segment VI. Findings were also consistent with uncomplicated cholelithiasis without biliary duct dilatation. The spleen, adrenals, pancreas, kidneys, and ovaries are all within normal limits (Figure 2).

Laboratory investigations were notable for leukocytosis with neutrophil predominance, as well as elevated inflammatory markers, including sedimentation rate, lactate dehydrogenase, C-reactive protein, and procalcitonin (Table 2). These findings raised a concern for intra-abdominal infection; therefore, the antibiotic regimen was escalated to intravenous meropenem at a dose of 1 g every eight hours for a total of 14 days.

**Table 2:** Laboratory values during her admission.

Test name	Results	Normal range
White blood cells	20.70/L	4-11/L
Neutrophils	19.60/L	7.5-10/L
Sedimentation rate	99 mm/hr	0-20 mm/hr
Lactate dehydrogenase	259 U/L	125-220 U/L
C-reactive protein	214 mg/L	5 mg/L
Procalcitonin	0.13 ng/mL	0.05 ng/mL

A multidisciplinary team involving obstetrics, general surgery, infectious disease, and neonatology was formed. The patient was counseled extensively regarding the management options, including surgical intervention via laparoscopic appendectomy versus a conservative approach. The risks and benefits of each strategy, including potential maternal and fetal mortality, were discussed in detail. After informed counseling, the patient declined surgical intervention and opted for conservative management.

Her obstetric ultrasonography demonstrated a singleton fetus in cephalic presentation with appropriate growth for gestational age, an estimated fetal weight of 2.3 kg, a normal amniotic fluid index of 15.77 cm, and normal Doppler studies. The patient was closely monitored for two weeks with twice-daily cardiotocography. Throughout her hospitalization, she remained clinically stable and asymptomatic, with reassuring fetal heart tracing and progressive improvement in laboratory parameters.

Following a 14-day observation period, the patient was deemed clinically stable, her blood cultures were negative, and she was discharged with a plan for readmission at 38-39 weeks of gestation for the induction of labor. In the event that an emergency cesarean section becomes necessary, the general surgery team will be involved. The patient was provided with clear instructions for returning to the emergency department and discharged in stable condition.

The patient was readmitted at 38 + 6 weeks of gestation for the induction of labor. On her admission, laboratory investigations were within normal limits. Repeated obstetric ultrasonography demonstrated a normal amniotic fluid index of 11 cm and normal Doppler studies. Induction of labor was initiated using a vaginal dinoprostone pessary (Propess, 10 mg). The first induction cycle was completed with reassuring fetal heart rate tracing; however, cervical examination revealed minimal progression, with dilatation of a fingertip. The patient subsequently underwent a 24-hour rest period prior to the second induction cycle.

Following the second cycle of induction, the patient progressed to 4 cm cervical dilation with 60% effacement and -3 station with intact membranes. She was transferred to the labor room, where she received epidural pain management. Artificial rupture of the membrane was done, which showed clear liquor. Labor progressed without complications, resulting in an uneventful vaginal delivery. Estimated blood loss was approximately 200 mL, and a second-degree vaginal tear was identified and repaired. She gave birth to a healthy baby boy who weighed 3.25 kg and had APGAR scores of 9 and 9 at five minutes. Postpartum observation continued for 48 hours, during which she remained asymptomatic. She was evaluated by the obstetric and general surgery team and was deemed fit for discharge, with planned outpatient follow-up with both teams.

**Figure 3:**
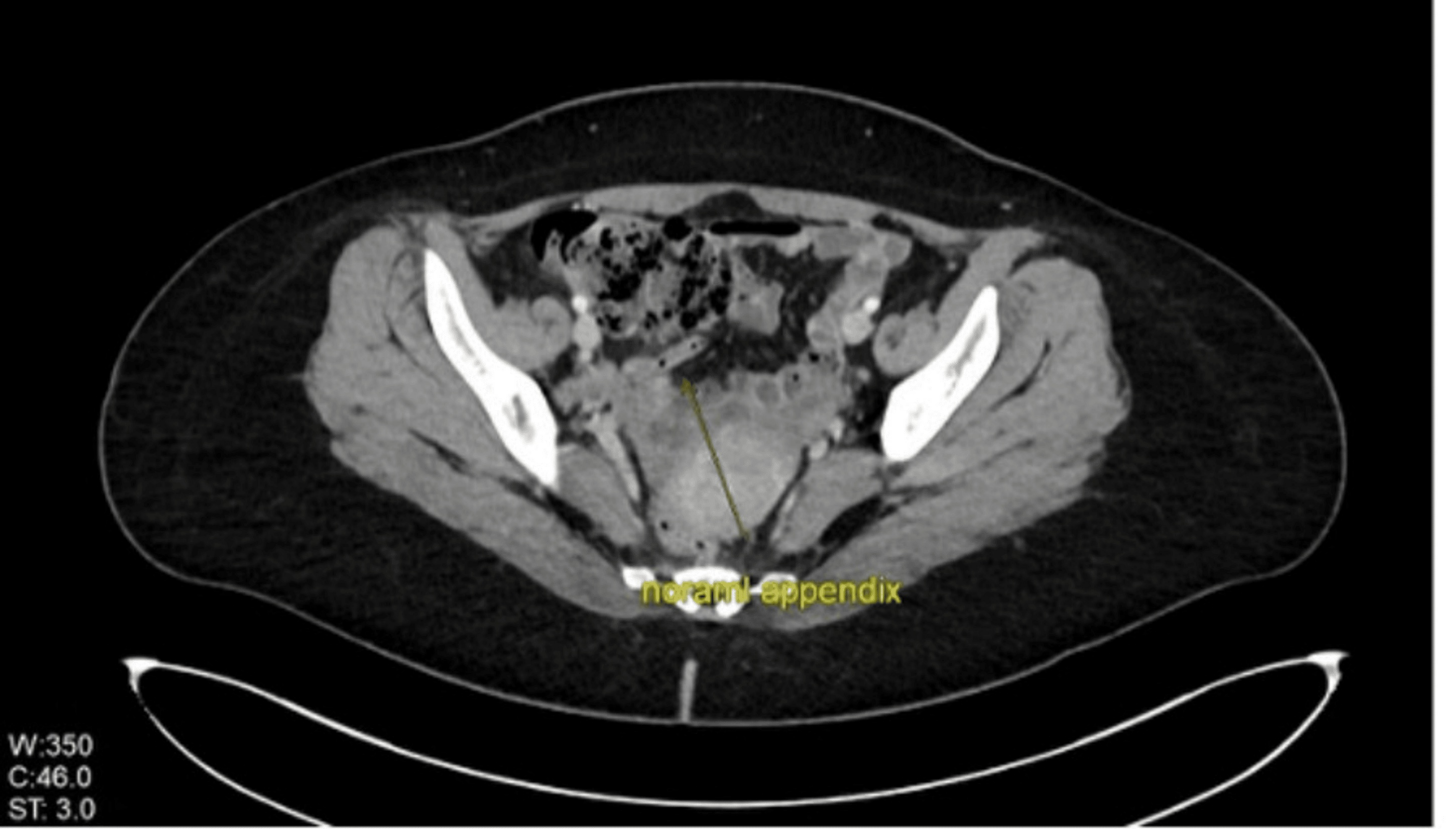
Computed tomography imaging of the abdomen. Computed tomography imaging of the abdomen during the follow-up of the patient, which showed a normal appendix with no signs of inflammation.

At subsequent outpatient follow-up, the patient remained asymptomatic and had resumed her daily life activities without limitations. Her computed tomography (CT) imaging demonstrated complete resolution of previous appendiceal inflammation, with no residual inflammatory changes (Figure 3). Follow-up inflammatory markers were within normal ranges. The patient was scheduled for a six-month follow-up and was provided with clear emergency department return instructions.

## Discussion

Acute appendicitis in pregnancy is the most common non-obstetrical emergency requiring immediate surgical intervention, with an incidence rate of 1:1,500 [1-7]. Due to the anatomical and physiological changes that occur in pregnancy, the diagnosis of acute appendicitis is challenging and requires a high index of clinical suspicion. These diagnostic challenges contribute to an increased risk of complications, including appendiceal perforation and its sequelae. These complications increase maternal and fetal mortality. The standard approach to diagnosing acute appendicitis in pregnancy consists of careful history taking, detailed physical examination, biomarkers, and proper imaging modalities to reduce the chances of delayed recognition. The preferred imaging modality is ultrasound. However, if ultrasound results are inconclusive, MRI may be employed. CT is reserved as a final option when other methods do not provide definitive information [6-19].

The optimal management of acute appendicitis in pregnancy remains a subject of ongoing debate. Surgical excision has traditionally been regarded as the standard treatment, specifically laparoscopic appendectomy. The surgical management is typically associated with reduced complications, as well as lower maternal and fetal mortality rates. However, it is important to consider the potential risks associated with surgery and anesthesia exposure in this patient population [20-23]. Recent studies suggest that conservative management can be considered a feasible alternative, avoiding surgical and anesthesia-related complications and facilitating faster recovery. Nonetheless, conservative management was associated with worse maternal and fetal outcomes, including increased maternal and fetal mortality primarily due to the sudden clinical deterioration and appendiceal rupture [25-27]. Phlegmonous appendicitis has been sparsely studied in pregnant populations; a systematic review including 42 studies was conducted in 2014 to assess the comparative efficacy of surgical intervention, specifically emergency appendectomy, versus conservative management strategies, which include antibiotic therapy or percutaneous drainage for phlegmonous appendicitis in adult and pediatric populations (no pregnant patients included). The incidence of surgical complications in adults is high, reaching up to 57%, with a 10% risk of necessitating intestinal resection. Conversely, all patients responded to antibiotic treatment [28]. Similarly, in a structured narrative review, Panahi et al. reviewed the management of phlegmonous appendicitis in adults, concluding that the conservative approach is preferable, provided that appropriate imaging and follow-up are implemented [29].

In a pregnant patient with phlegmonous appendicitis, conservative management might be considered rather than routinely pursuing surgical management. This strategy follows a stepwise, multidisciplinary approach involving obstetricians, general surgeons, and neonatologists. This approach relies on comprehensive counseling regarding the available management strategies, including a detailed discussion of the benefits and potential drawbacks inherent to each option. If the patient prefers conservative management, a detailed assessment of maternal and fetal well-being will be conducted using comprehensive ultrasound imaging, Doppler flow studies, and daily cardiotocography to ensure optimal monitoring of both maternal and fetal health. The general surgical team is tasked with the critical evaluation of diagnostic imaging and biomarkers indicative of appendicitis, employing evidence-based methodologies to determine the appropriate clinical intervention. If the patient maintains clinical and laboratory stability throughout the observation period, she may be discharged without any alterations to the timing or mode of delivery. Acute appendicitis during pregnancy represents a significant diagnostic dilemma, mainly due to its atypical clinical presentation. Further research is urgently needed, particularly focusing on the development of novel biomarkers facilitating a more accurate and timely recognition of appendicitis in the pregnant population. Additionally, conservative management should be critically and rigorously evaluated to strengthen the evidence base and optimize maternal and fetal outcomes in this unique clinical context.

## Conclusions

Acute appendicitis in pregnancy is the most prevalent non-obstetrical emergency that requires immediate surgical intervention. The management of acute appendicitis is contentious. A conservative approach in selected cases of complicated appendicitis might prove to be a reasonable alternative during pregnancy. This evolving perspective highlights the need for careful consideration of patient-specific factors and the potential risks and benefits associated with each treatment modality. The outcomes of this case are insufficient to draw definitive conclusions in terms of conservative management of acute appendicitis. More cases and studies are required to develop evidence-based guidelines to optimize outcomes for both the mother and the fetus.
